# Epigenetic regulation of cancer stem cell plasticity and relapse competence in EGFR-mutant lung cancer

**DOI:** 10.3389/fonc.2026.1981354

**Published:** 2026-10-19

**Authors:** Xiangxiang Sun, Shengshan Xu, Qian Guo

**Affiliations:** 1 Jiaxing First Hospital, Jiaxing, Zhejiang, China; 2 Department of Thoracic Surgery, Jiangmen Central Hospital, Jiangmen, Guangdong, China; 3 Zhongnan Hospital of Wuhan University, Wuhan, Hubei, China

**Keywords:** cancer stem cells, drug-tolerant persister cells, EGFR-mutant lung cancer, epigenetic plasticity, lineage plasticity, minimal residual disease, osimertinib, RNA epigenetics

## Abstract

Cancer stem cell (CSC) plasticity is increasingly linked to therapy resistance, yet CSC identity is still often inferred from persistence, stemness markers, or short-term regenerative assays. EGFR-mutant non–small-cell lung cancer is a useful setting in which to examine this problem because residual states can be sampled before stable resistance is established. Here, we focus on how chromatin remodeling, DNA methylation, histone regulation, RNA epigenetics, adaptive signaling, metabolism, and the immune microenvironment influence stem-like behavior during EGFR-targeted therapy. We assess the literature using four evidence levels: state association, functional enrichment, post-treatment durability, and lineage-resolved acquisition of durable CSC function. The available data strongly support epigenetic regulation of therapy-persistent and resistant states, and several models show functional overlap with CSC-associated phenotypes. What remains largely missing is direct evidence that a prospectively identified persister lineage later acquires serially propagating CSC function. Minimal residual disease may therefore represent a period in which relapse-competent states are present but not yet irreversibly established.

## 1 Introduction

Cancer stem cells matter clinically because of what they can do: self-renew, regenerate heterogeneous tumor populations, and sustain recurrence. In EGFR-mutant non–small-cell lung cancer (NSCLC), first-line osimertinib produces deep responses and prolongs survival, but residual disease often remains and may later seed heterogeneous relapse (1, 2). This makes EGFR-targeted therapy a useful setting in which to ask a more specific CSC question: which surviving cells retain, or later acquire, durable tumor-regenerating capacity, and what epigenetic mechanisms support that capacity? CSC identity is therefore best judged by function. Durable self-renewal, generation of heterogeneous progeny, and long-term tumor propagation are more informative than any single marker or pathway signature (3–5). Current CSC models also allow for reversible state transitions rather than a permanently fixed stem-cell compartment (6). In lung cancer, ALDH-positive populations can be enriched for EGFR-TKI resistance (7), yet tumor-initiation capacity and treatment resistance can also separate experimentally (8). Thus, drug resistance, EMT, quiescence, or expression of SOX2 and ALDH1A1 should be treated as CSC-associated features, not as substitutes for CSC identity.

Drug-tolerant persister (DTP) cells are useful because they capture an early phase of adaptation, before stable resistance dominates. The original DTP study described a reversible chromatin-associated state (9), while later work showed that resistant populations may arise from pre-existing clones or from drug-tolerant intermediates (10). Barcoding and lineage-tracing experiments add another layer: persister lineages can differ before treatment and continue to change during EGFR inhibition (11, 12). Patient single-cell data likewise show a heterogeneous survivor population rather than a uniform residual state (13). DTP biology itself has been reviewed extensively (14); here, the narrower issue is how epigenetic regulation changes CSC-related state competence and what evidence is needed before persistence can be linked to relapse-propagating CSC behavior. We use EGFR-mutant NSCLC as a tractable clinical model, not because the hierarchy is disease-specific. EGFR-TKI treatment provides a relatively clear sequence from deep response through residual disease to acquired resistance, with serial tissue/ctDNA sampling, patient-derived models, and single-cell datasets available across this trajectory.

## 2 Dynamic CSC identity in a treatment-perturbed residual ecosystem

DTP status describes survival under treatment; minimal residual disease (MRD) describes residual tumor burden at the tissue or patient level. Neither term defines a CSC. In practice, a residual tumor may contain reversible DTPs, cycling persisters, pre-existing stem-like lineages, and lineage-plastic cells at the same time (11, 12), a degree of heterogeneity also seen in patient single-cell data (13).

We use a four-level hierarchy to judge therapy-associated CSC claims. Level A requires a molecular or phenotypic state linked to stemness. Level B requires direct regenerative evidence in the defined population, such as clonogenic regrowth, sphere/organoid formation, or tumor initiation. Level C requires Level B function to persist after drug withdrawal and recovery, preferably on repeat testing or rechallenge; molecular persistence alone is insufficient. Level D additionally requires prospective labeling or lineage tracing linking a defined therapy-persistent population to durable CSC function. Each study receives the highest level directly demonstrated within the same experimental system; evidence from separate models is not combined. These are levels of evidence, not sequential cellular states, disease stages, or patient categories. These levels answer four questions: does a cell resemble a stem-like state, function like one, retain that function after treatment pressure is removed, and where did the cell come from? A single tumorsphere assay can support Level B but not Level C; Level D cannot be assigned without prospective lineage information. Evidence grades follow the experiment rather than the cell label. **Figure 1** summarizes the hierarchy, and **Table 1** gives the minimum criteria and representative applications.

**Figure 1:**
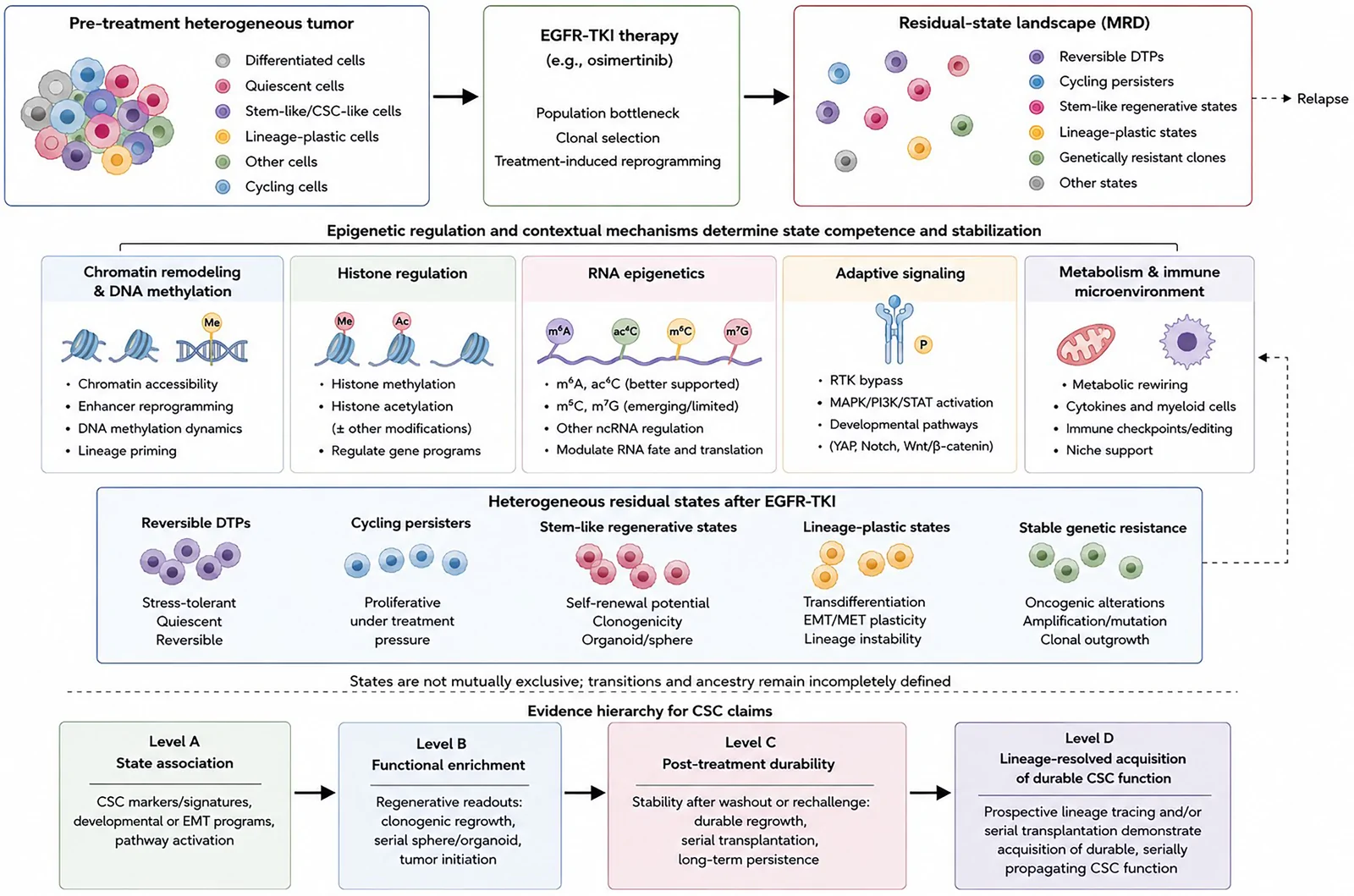
Conceptual model of therapy-perturbed residual states and evidence hierarchy for cancer stem cell claims in EGFR-mutant lung cancer. EGFR-TKI therapy imposes selective pressure on a heterogeneous tumor population, with both pre-existing cellular states and treatment-induced reprogramming contributing to the residual-state landscape. Epigenetic regulation, adaptive signaling, metabolic rewiring, and the immune microenvironment influence the competence, stabilization, and fitness of residual cells. Reversible drug-tolerant persisters (DTPs), cycling persisters, stem-like regenerative states, lineage-plastic states, and genetically resistant populations may coexist after treatment; these categories are not necessarily mutually exclusive, and direct lineage relationships among them remain incompletely resolved. Accordingly, a stem-like regenerative state is represented as one possible outcome of therapy-associated plasticity rather than an obligatory intermediate between DTP persistence and acquired resistance. The evidence hierarchy distinguishes four levels of support for CSC-related claims: Level A, molecular or phenotypic state association; Level B, functional enrichment for regenerative capacity; Level C, persistence of previously demonstrated regenerative function after treatment withdrawal and recovery; and Level D, lineage-resolved acquisition of durable CSC function. This framework separates treatment persistence and stem-like resemblance from direct evidence of durable, relapse-propagating CSC identity. The A-D levels represent evidence strength rather than sequential biological states or patient categories. Level C requires persistence of Level B function after drug withdrawal and recovery, whereas Level D additionally requires prospective lineage evidence.

**Table 1:** Operational criteria for levels A-D and evidence audit of therapy-associated stem-like states in EGFR-mutant lung cancer.

Part A. Operational criteria for the A-D evidence hierarchy					
Level	Minimum measurable criteria		Timing/required validation		Key boundary
A	Molecular or phenotypic stem-like state (markers/signatures, developmental/EMT programs, or single-cell state)		Measured during or after therapy; no direct CSC functional assay required		Does not establish CSC function
B	Direct regenerative function in the defined population (clonogenic regrowth, sphere/organoid formation, or tumor initiation)		Reproducible functional assay at the measured treatment/resistant state		Does not establish post-withdrawal durability or ancestry
C	Level B function persists after drug withdrawal and recovery		Repeat functional testing after washout; preferably recovery/rechallenge		Molecular persistence alone is insufficient; ancestry remains unproven
D	Prospectively defined therapy-persistent lineage acquires durable CSC function		Prospective lineage tracing plus Level C functional validation through recovery/relapse		Endpoint resistant populations without ancestry do not meet Level D

Part B. Representative evidence audit					
Study	Model/stage	Mechanism	CSC-related readout	Evidence grade	Interpretation
Sharma et al. (9)	Early drug tolerance	Reversible chromatin-associated DTP state	Survival/reversibility; no durable CSC assay	A	Foundational persistence evidence; DTP identity is not CSC identity.
Criscione et al. (19)	Osimertinib DTP	ATAC/RNA remodeling; BRD4/TEAD	State signatures; washout transcriptional carryover	A (molecular carryover noted; not Level C)	Strong DTP-stage epigenetic evidence; washout transcriptional carryover was observed, but persistent CSC-related function was not tested.
Maynard et al. (13)	49 patient biopsies; targeted therapy	scRNA-seq of residual/progressive disease	Alveolar-regenerative/primitive residual state; outcome-associated features	A (patient data)	Clinically anchors state association; CSC function and ancestry were not tested.
Liang et al. (22)	DTP/residual/resistant	ZNF263-DNMT1-EGFR methylation	Reduced residual disease/regrowth	A	Strong residual-state mechanism; no CSC regenerative assay.
Fang et al. (28)	EGFR-TKI-resistant NSCLC	p300-H3K27ac-NAT10-ac4C-FATP4/CPT1A	Metabolic fitness; drug resistance	A (CSC question)	Cross-layer mechanism; direct CSC function not established.
Jeong et al. (15)	EGFR-mutant MPE; TKI sensitive/resistant	scRNA-seq; epithelial and immune plasticity	Precursor-like epithelial enrichment; HLA class II association with PFS	A (patient data)	Links cellular state with TKI response/PFS; no direct self-renewal or ancestry test.
Yi et al. (16)	Established osimertinib-resistant	HER3-PAK2-β-catenin	Cancer stem-like properties and resistance	B (resistant endpoint)	Functional convergence; durability and ancestry unresolved.
Daley et al. (50)	DTP, spheroid, *in vivo*	SOS1 adaptive signaling	Spheroids, tumor initiation, tumorigenesis	B	Strong functional bridge; durability and lineage-resolved CSC acquisition remain unproven.

For clinical interpretation, Level A is most directly supported by patient profiling. Maynard et al. identified an alveolar-regenerative/primitive program in 49 biopsies from 30 patients during targeted therapy (13). Jeong et al. found precursor-like cells enriched in TKI-resistant pleural effusions, with HLA class II expression associated with PFS (15). These data link state to outcome but not CSC function. Yi et al. paired relapse-associated HER3/PAK2/β-catenin activity and shorter PFS with experimental cancer stem-like function, providing a Level B bridge (16). Level C requires that function to persist after withdrawal and recovery; Level D adds prospective ancestry. Routine patient scRNA-seq alone establishes neither.

The hierarchy is not disease-specific. In HER2-positive breast cancer, Chang et al. identified G0-like pre-DTP states, patient signatures associated with poorer TKI response, and barcode-defined DTP ontogeny (17). Shah et al. showed lapatinib enrichment of JAGGED1-high/NOTCH-active cells with mammosphere and tumor-initiating capacity; high membrane JAGGED1 also correlated with poorer overall survival (18). These data still do not demonstrate lineage-resolved acquisition of durable CSC function.

## 3 Epigenetic control of CSC-related state competence and stabilization

### 3.1 Chromatin competence and DNA/histone-state regulation

Chromatin regulation is relevant to CSC biology because it can change which phenotypic states remain accessible during therapy. In osimertinib DTPs, integrated RNA-seq and ATAC-seq identified dynamic regulatory remodeling together with BRD4 and TEAD dependencies (19). Established osimertinib-resistant models show a different vulnerability: mSWI/SNF complexes are required to maintain resistance-associated programs, and disruption of SMARCA4/SMARCA2 can restore drug sensitivity (20). They do not, however, imply a single linear path from persistence to CSC identity. Both the pretreatment state and the response to drug exposure influence which CSC-related phenotypes emerge. ASCL1, for example, promotes osimertinib tolerance only in permissive cellular contexts, and related DTP-like states may already be detectable before therapy (21). ZNF263 recruits DNMT1 to the EGFR promoter and is reduced in DTP, resistant, and clinical residual samples; restoring ZNF263 improves EGFR-targeted responses and limits regrowth (22). HDAC5 is induced early after osimertinib exposure, and its depletion or inhibition increases histone acetylation while reducing subsequent resistant-cell emergence (23).

In EGFR-mutant models, EZH2 loss lowers H3K27me3 at the MET promoter and facilitates MET-dependent TKI resistance (24). In other drug-resistant NSCLC models, by contrast, combined EZH2/G9a activity promotes tumorsphere formation and malignant phenotypes through SMAD4/ERK/c-Myc signaling (25). For CSC biology, the relevant question is whether EZH2-dependent regulation changes durable regenerative capacity, not simply whether it changes resistance.

### 3.2 RNA epigenetics and cross-layer control of stem-like fitness

The cLMNB1-FGFR4 study requires a different interpretation. cLMNB1 promotes c-Cbl-dependent ubiquitination and degradation of FGFR4 and thereby increases osimertinib sensitivity. In resistant cells, METTL3/YTHDF2-dependent m6A accelerates cLMNB1 degradation, removing this sensitizing effect; an m6A-site-mutated cLMNB1 is more stable and more active in resistant models (26). Thus, cLMNB1 itself overcomes resistance, whereas m6A-dependent loss of cLMNB1 favors resistance. Broader evidence supports transcript-specific RNA-epigenetic effects. METTL3-mediated m6A stabilizes SNHG17, which recruits EZH2 to repress LATS2 and promotes gefitinib resistance (27). NAT10-dependent ac4C stabilizes FATP4 and CPT1A transcripts and supports fatty-acid metabolism and EGFR-TKI resistance; NAT10 transcription is promoted by p300-H3K27 acetylation (28). These studies link RNA modification with chromatin regulation and metabolism, but they mainly examine established resistant states and do not establish Level C durability or Level D ancestry.

### 3.3 Adaptive signaling as an effector of epigenetic competence

Adaptive signaling should be separated conceptually from epigenetic regulation, even though the two often interact. YAP/TEAD supports apoptosis-resistant persistence, and CRISPR screens confirm Hippo-pathway dependence in EGFR-mutant persisters (29, 30). Developmental signaling follows a similar pattern: Takahashi et al. showed that NOTCH1 and Notch target genes are increased in osimertinib DTPs and that gamma-secretase inhibition impairs persistence (31), while Notch3-dependent β-catenin signaling supports adaptive persisters (32). These pathways overlap with stemness programs, but pathway activation by itself remains Level A evidence.

PAK2-β-catenin signaling increases cancer stem-like properties in established osimertinib-resistant cells (16), while protective autophagy maintains SOX2/ALDH1A1-associated stem-like phenotypes during osimertinib stress (33). Their limitation is temporal: because the measurements are made in resistant or treatment-adapted endpoints, they cannot show when the regenerative phenotype first appeared or whether it arose within a DTP lineage.

## 4 Selection, induction, and branching state transitions

Hata et al. showed that EGFR-TKI resistance can arise through selection of pre-existing resistant clones or through evolution from initially drug-tolerant cells (10). Expressed barcoding and lineage tracing similarly show that persister lineages can carry distinct pretreatment programs and still adapt during therapy (11, 12). Clinical observations fit a branching model better than a single persister-to-CSC route. Histologic transformation is another possible outcome of plasticity, not evidence for a required CSC intermediate. Baseline RB1/TP53 alterations increase the risk of small-cell transformation, and clonal reconstruction suggests that small-cell and adenocarcinoma trajectories may diverge before therapy (34, 35). These observations point to loss of lineage constraint; they do not require prior acquisition of CSC identity. Persistence also gives tumor cells time to acquire genetically stabilized resistance. Therapy-induced APOBEC3A increases mutagenesis in drug-tolerant cells and accelerates acquired resistance (36), while AXL-associated error-prone replication links cycling persisters to mutational evolution (37). This is consistent with a branching model in which pretreatment state, drug-induced reprogramming, microenvironmental selection, and later genetic fixation all contribute to relapse.

## 5 Epigenetic memory and relapse competence

An epigenetic change observed during drug exposure should not automatically be called memory. Criscione et al. found that selected DTP-associated transcriptional programs persisted during washout (19). That result is important, but without longitudinal evidence that the underlying regulatory state and function are retained, ‘transcriptional carryover’ is a more precise description than stable epigenetic memory. Nor does a stable tendency to persist necessarily imply treatment-induced memory. The ‘chance to persist’ can be heritable before treatment and is associated with IRS1 phosphorylation (38), showing that stable cellular propensity can predate therapy and need not be carried by a canonical epigenetic mark. YAP/TEAD-dependent dormancy is also reversible after pathway suppression (29). Prolonged survival and extensive transcriptional remodeling, therefore, are not sufficient evidence of permanent identity. For a Level C CSC claim, the regenerative phenotype itself should persist after drug withdrawal, preferably through renewed proliferation and rechallenge. Serial organoid or sphere regeneration carries more weight than a single rebound assay, and repeated tumor propagation carries more weight still. Ideally, molecular and functional persistence should be measured in the same lineage. Otherwise, a durable molecular signature cannot be assumed to encode durable CSC behavior.

## 6 Metabolic and immune regulation of residual-state fitness

Inosine accumulates in EGFR-TKI-resistant cells, osimertinib DTPs, and clinical residual tumors; inosine-A2A receptor signaling increases oxidative phosphorylation and resistance, while secreted inosine promotes M2-like macrophage reprogramming (39). Patient-derived single-cell analysis has also identified GSTA1-high osimertinib DTPs with enhanced drug metabolism and macrophage interactions through PROS1-AXL signaling, further linking residual-cell metabolism with an immunosuppressive microenvironment (40). More broadly, one-carbon metabolism illustrates how nutrient availability can couple methyl-donor supply, epigenetic programming, and T-cell fitness within the tumor microenvironment (41). In lung adenocarcinoma models outside the EGFR-TKI persister setting, PANX2-driven disulfidptosis promotes extracellular ATP/P2X7R-dependent antitumor immune infiltration (42), whereas IER3 supports NSCLC survival by suppressing ferroptosis through AKT/GSK3β/NRF2 signaling (43). These studies broaden the metabolic, redox, and immune context of residual-state fitness, but the latter examples do not establish CSC function or persister ancestry. Lipid metabolism provides a more direct bridge to stem-like behavior. SCD1 supports lung cancer stemness through β-catenin and YAP/TAZ signaling (44), and SCD1 inhibition suppresses cisplatin-enriched spheroid formation and CSC-associated phenotypes (45). In the EGFR-TKI setting, NAT10-ac4C stabilization of FATP4/CPT1A also supports fatty-acid metabolism (28). Read together with single-cell evidence of precursor-like states and immune remodeling in resistant pleural effusions (15), these data suggest that metabolic and microenvironmental conditions can favor regenerative or lineage-plastic states without identifying their ancestry.

## 7 Therapeutic implications: intercept plasticity by state and timing

Therapeutic timing matters because the relevant dependency changes with cell state. Early HDAC5 inhibition can reduce adaptive-state formation (23), and YAP/TEAD-directed approaches suppress persister survival during active EGFR inhibition (30, 46). Once persistence or resistance is established, a different set of dependencies becomes relevant. mSWI/SNF inhibition can collapse resistance-associated chromatin programs (20); NAT10-ac4C targeting disrupts a post-transcriptional metabolic dependency (28); and inosine-A2A blockade interferes with both tumor-cell oxidative phosphorylation and macrophage support (39). These are more accurately described as state-directed strategies than CSC-directed therapies, because they target features of the adapted population rather than a validated CSC-specific dependency.

A third strategy is direct elimination of residual cells with relapse potential. TROP2-directed CAR-T cells can eradicate osimertinib-induced DTPs and extend relapse-free survival *in vivo* (47), while CD24 blockade enhances macrophage-mediated clearance of EGFR-TKI-treated residual cells (48). TROP2 antibody-drug conjugate combinations also suppress DTP emergence and delay relapse (49). These are still residual-cell strategies unless durability and lineage criteria are met. SOS1 inhibition is more directly relevant to the CSC question because it reduces DTP emergence together with spheroid growth, tumor-initiating frequency, and *in vivo* tumorigenesis, providing a strong Level B bridge toward relapse competence (50). Biomarkers should distinguish residual burden from residual-cell state. ctDNA-defined MRD can precede radiographic recurrence, but it does not reveal the phenotype of the cells that remain (51). Paired molecular profiling at first-line osimertinib progression also confirms marked heterogeneity in the resistant compartment (52). Clinically, a marker of reversible persistence may call for a different intervention from a marker associated with post-withdrawal regeneration. The aim is therefore not simply to detect residual disease, but to identify which residual states are still reversible, which are being stabilized, and which retain durable regenerative capacity.

## 8 Evidence gaps and an experimental roadmap

A convincing test of therapy-induced CSC acquisition has to connect ancestry, regulatory state, durable function, and relapse in the same lineage. High-complexity barcoding has shown that rare EGFR-TKI-resistant clones can pre-exist treatment (53), while expressed barcoding resolves persister ancestry and state behavior at high resolution (11, 12). ClonMapper-type approaches add an important practical advantage: defined clones can be recovered for subsequent functional testing (54).

A workable design would barcode cells before treatment, identify DTPs prospectively during active osimertinib exposure, and sample matched lineages through acute response, DTP/MRD, withdrawal, proliferative recovery, rechallenge, and relapse. Paired scRNA-seq and scATAC-seq could then be complemented, when the mechanism warrants it, by DNA methylation, histone, or RNA-modification measurements. Functional testing should include serial organoid or sphere regeneration and, where feasible, limiting-dilution tumor initiation followed by serial transplantation. Timing is equally important for causal testing. Candidate epigenetic regulators should be perturbed during the DTP window while leaving enough viable cells to separate altered fate from nonspecific cytotoxicity. If those surviving cells recover bulk proliferation but lose serial tumor-propagating capacity, the experiment would connect a treatment-stage regulatory mechanism with later regenerative competence. Mapping relapse tissue back to the original barcode would then help distinguish pre-existing CSC selection, therapy-induced CSC acquisition, and parallel lineage evolution. Level D therapy-induced CSC acquisition should therefore be reserved for experiments in which a prospectively defined persistent lineage acquires a treatment-associated regulatory state, retains the relevant molecular or functional phenotype after withdrawal, and later demonstrates durable self-renewal with heterogeneous tumor propagation. At present, the literature supports epigenetic regulation of persistence and functional convergence more strongly than lineage-resolved DTP-to-CSC conversion.

## 9 Discussion and conclusion

A useful way to read the current evidence is as four interacting layers through which epigenetic plasticity may contribute to CSC-driven relapse without implying a fixed CSC compartment. State competence concerns which trajectories are accessible in a given lineage and chromatin context (19, 21). State stabilization concerns how selected programs are maintained through DNA/histone regulation, chromatin remodeling, and RNA modification (22, 28). State memory asks whether these regulatory or functional changes persist after drug withdrawal (19, 24, 38). Ecological fitness describes whether a regenerative state can survive and compete within a residual ecosystem shaped by metabolism, nutrient competition, and immune interactions (39–41). The boundaries between these layers are not rigid, but the distinction is useful experimentally. The evidence hierarchy provides a separate standard for what can be claimed about CSC identity. Level A indicates molecular resemblance; Level B demonstrates regenerative function; Level C requires Level B function to persist after withdrawal and recovery; and Level D adds prospective lineage evidence linking that durable function to a therapy-persistent lineage. Several interpretive shortcuts follow from this distinction: drug survival is not CSC identity, marker enrichment is not self-renewal, functional enrichment is not memory, and durable function alone does not establish ancestry. Most published studies fall within Levels A and B. Molecular carryover after washout has been reported in selected models, but few studies meet the full Level C criteria and direct Level D evidence remains scarce.

This distinction changes the translational question. Rather than asking which DTP marker should be targeted, the more useful question is which regulatory process a relapse-propagating state depends on, and when that dependence is present. MRD is particularly relevant because some treatment-associated states may still be reversible before durable relapse behavior develops. Prospective lineage tracing, longitudinal epigenetic profiling, withdrawal and rechallenge, and rigorous CSC functional assays are needed to separate selection of a pre-existing regenerative lineage from induction of a durable relapse-propagating state. The same studies should also reveal which epigenetic mechanisms are realistic targets for preventing CSC-driven recurrence.
